# Digital mental health interventions for adolescents and young people (10–24 years) in Africa: A protocol for a systematic review of mental health outcomes, engagement, and equity considerations

**DOI:** 10.12688/wellcomeopenres.24117.1

**Published:** 2025-07-04

**Authors:** Aggrey Gisiora Mokaya, Nondumiso Michelle Dlamini, Syethemba Nkosi, Mpilonhle Thembinkosi Nzuza, Thandeka Smith, Grace Nduku Wambua, Xanthe Hunt, Alan Stein, Nothando Ngwenya

**Affiliations:** 1 Social Science, Africa Health Research Institute, Durban, KwaZulu-Natal, 4001, South Africa; 2 University of KwaZulu-Natal College of Health Sciences, Durban, KwaZulu-Natal, South Africa; 3 University of Oxford, Oxford, England, UK

**Keywords:** Digital mental health; mHealth; eHealth; Adolescents; Young adults; Youth mental health; Africa; Health equity; Mobile health interventions; Psychological well-being; Systematic review.

## Abstract

**Background:**

Adolescents and young people (AYP) aged 10–24 years in Africa experience a high burden of mental health disorders but face significant barriers to accessing care, including a severe shortage of mental health professionals, stigma, and limited integration of mental health services into primary healthcare. Digital mental health interventions (DMHIs) offer a promising avenue to bridge these gaps by providing accessible, scalable, and potentially equitable support. However, little is known about the effectiveness, acceptability, and equity impacts of these interventions among African AYP.

**Objective:**

This systematic review aims to identify, characterize, and synthesize evidence on DMHIs targeting adolescents and young people in Africa, focusing on mental health outcomes, engagement, feasibility, and equity considerations.

**Methods:**

We will search PubMed, Scopus, Web of Science, and EBSCOhost databases, as well as the PsyberGuide repository, for empirical studies published between January 1, 2015, and April 3, 2025. Eligible studies must evaluate a digital mental health intervention among AYP aged 10–24 years living in Africa. Study designs will include randomized controlled trials, quasi-experimental studies, observational studies, and mixed-methods studies. Outcomes of interest include mental health symptom reduction (depression, anxiety, stress), psychological well-being, engagement, feasibility, acceptability, and equity-related factors such as gender inclusivity and digital access. Risk of bias will be assessed using RoB 2 and ROBINS-I tools, and evidence quality will be rated using the GRADE approach. Quantitative synthesis will be conducted where feasible, otherwise narrative synthesis will be employed.

**Conclusion:**

This review will provide a comprehensive synthesis of available evidence on DMHIs for adolescents and young people in Africa, offering critical insights into their effectiveness, feasibility, and contribution to promoting mental health equity. The findings aim to inform the development, implementation, and policy integration of digital mental health strategies tailored to young populations in diverse African contexts.

## Plain Language Summary

Mental health challenges such as depression, anxiety, and stress are common among adolescents and young people in sub-Saharan Africa. However, access to mental health services is often limited due to stigma, a shortage of trained professionals, and barriers related to cost, distance, and awareness. Digital tools, like mobile phone apps, SMS-based support, or online counselling platforms, could help improve access to mental health care for young people. This review aims to find and summarize existing research studies that have tested digital mental health interventions among adolescents and young people aged 10–24 years in sub-Saharan Africa. We will focus on studies that looked at how effective these interventions are, how acceptable and feasible they are to users, and whether they address inequalities in access to care. The findings will help researchers, health workers, and policymakers understand what has been tried, what works, and what gaps remain. This information can guide the development of better mental health support for young people in the future.

**PROSPERO Registration:**

CRD420251025765.

**Amendments:**

This protocol does not represent an amendment of a previously completed or published protocol. Any important future amendments, including updates to funding information, methodological changes, or review status, will be documented directly in the PROSPERO record with dated revision notes.

## Introduction

### Rationale

Adolescents and young people (AYP) in Africa, aged 10 to 24 years, experience unique vulnerabilities due to the interplay of biological, psychological, and socio-environmental factors that influence their mental well-being ^1– 5^. As a result, the burden of mental health disorders among African AYPs is high, with median prevalence rates ranging from 26.9% to 40.8% for depression, anxiety disorders, and emotional or behavioral problems ^6^. Despite the growing demand for mental health interventions ^6– 10^, access to care remains limited due to a severe shortage of mental health professionals—averaging just 0.05 psychiatrists per 100,000 people in Africa, compared to over 10 per 100,000 in high-income countries ^3, 11^. While task-shifting has been widely adopted to address this gap, its effectiveness depends on proper implementation ^12– 14^. In some cases, task-shifting can improve access to care, but challenges in training, supervision, and support may lead to suboptimal outcomes ^15^. Additionally, task-shifting may not always be suitable for certain contexts, potentially compromising the quality and effectiveness of care.

Digital health technologies encompass a broad range of electronic tools, platforms, and systems designed to improve healthcare delivery, patient outcomes, and overall health management ^16, 17^. These technologies include telemedicine, mobile health applications, wearable devices, electronic health records, artificial intelligence-driven diagnostics, and remote monitoring tools ^16^. By leveraging digital innovations, they enhance accessibility, efficiency, and personalization in healthcare, bridging gaps in service delivery and supporting data-driven decision-making. Specifically, digital mental health interventions (DMHIs) —including mobile applications, telepsychiatry, internet-based cognitive behavioural therapy (iCBT), and artificial intelligence-driven mental health tools—have emerged as promising strategies for expanding access to quality care in settings where traditional mental health services remain scarce ^18, 19^. These technologies offer significant potential in Africa, where young people often experience barriers to mental health care, including stigma surrounding mental illness ^20– 22^, inadequate integration of mental health into primary healthcare systems ^23– 25^ and the aforementioned severe shortage of mental health professionals, among others. By leveraging digital platforms, these interventions can provide young people with tailored, accessible, and scalable mental health support that would otherwise be difficult to obtain ^26– 29^.

Health equity in digital mental health refers to the fair and just distribution of mental health benefits, ensuring that young people—regardless of their socioeconomic status, gender, geographic location, or digital literacy—can access and engage with these innovations ^30, 31^. Without explicit consideration of equity, digital interventions risk reinforcing existing disparities rather than addressing them ^32^. These disparities contribute to a digital divide, which encompasses more than just infrastructure and internet connectivity. It is also shaped by gendered disparities in mobile phone ownership, the affordability of data and devices, language and literacy barriers, and cultural attitudes toward mental health ^33, 34^. This divide significantly influences how young people engage with digital interventions and determines whether these technologies can effectively reach the most marginalized populations.

Moreover, Africa is diverse and rapidly evolving, with significant variation in economic development, healthcare infrastructure, and the adoption of digital technologies ^35^. While previous reviews have examined digital mental health interventions in low- and middle-income countries, there remains a gap in understanding how these interventions function for young people on the continent ^26^. Africa is experiencing an accelerated deployment of mobile phones, smart devices, the Internet of Things (IoT), artificial intelligence, and augmented reality, all of which are increasingly being piloted and tested for integration into health systems to address unmet health needs ^36, 37^. These technological advancements present both opportunities and challenges. On one hand, they offer innovative ways to deliver mental health support to young people; on the other, they risk excluding those who lack digital access or literacy, thereby widening health inequities.

This systematic review applies the World Health Organization’s evaluation framework for digital health interventions, assessing their acceptability and feasibility, as well as their implications for gender, equity, human rights, and resource requirements ^38^. By synthesizing evidence across these dimensions, this review seeks to provide a nuanced understanding of how digital mental health technologies are being implemented for young people in Africa and whether they contribute to improving equity in mental health care. The findings will offer valuable insights for policymakers, practitioners, and researchers working to ensure that digital mental health solutions are not only effective but also accessible, inclusive, and responsive to the diverse needs of young people across the region.

### Objectives

To systematically review the evidence on digital mental health interventions targeting adolescents and young people (10–24 years) in Africa, with the following specific objectives:

1. Identify and characterize DMHIs implemented in African settings.
2. Evaluate mental health outcomes associated with these interventions.
3. Assess acceptability, feasibility, and engagement.
4. Examine equity and implementation considerations, including gender, access, and digital literacy.

## Methods

### Eligibility criteria

**Participants:** Adolescents and young people aged 10 to 24 years living in Africa. Studies including broader age groups will be included only if disaggregated data for AYP are reported.

**Interventions:** Digital mental health interventions (DMHIs) including:

- mHealth (SMS, mobile apps)
- eHealth platforms
- CCBT (Computerized Cognitive Behavioural Therapy)
- Teletherapy / telepsychiatry
- Serious games
- AI-powered mental health tools
- Virtual Reality/ Augmented Reality applications

**Comparators:**

Any comparator, encompassing:

- Active controls (e.g., face-to-face counselling)
- Passive controls (e.g., waitlist, treatment as usual)
- No comparator (for single-arm or feasibility studies)

**Outcomes:**

- Main: Depression, anxiety, psychological well-being, stress, emotional regulation, engagement, feasibility.
- Other: Equity of access, digital literacy, gender inclusivity, financial accessibility.

**Study Designs:**

- RCTs, quasi-experimental, cohort, case-control, cross-sectional, observational, mixed-methods.

**Exclusions:**

- Studies outside Africa
- Protocols, editorials, opinion pieces, literature reviews
- Studies with no evaluation of a digital mental health intervention
- Studies before January 1, 2015

### Information sources

The systematic review will draw on a range of information sources to comprehensively identify both published and grey literature on digital mental health interventions (DMHIs) for adolescents and young people (AYP) in Africa.

***Electronic databases***

The following databases will be searched systematically:

- PubMed
- Scopus
- Web of Science
- EBSCOhost (including PsycINFO, Academic Search Complete, CINAHL, and Global Health subsets where accessible)

Each database will be searched for records published between January 1, 2015, and April 3, 2025. Filters will be applied to restrict results to English-language, peer-reviewed empirical studies. Non-empirical content (e.g., editorials, protocols, reviews) will be excluded during screening.

***App repository: psyberGuide***

In addition to academic databases, PsyberGuide, a curated digital mental health app repository, will be included to identify real-world DMHIs potentially targeting AYP. Apps listed on PsyberGuide as of April 2025 will be cross-referenced with empirical evaluations in PubMed, Scopus, Web of Science, and Google Scholar. Only those meeting inclusion criteria—based on population, digital delivery, geographic setting, and study design—will be retained.

***Grey literature and other sources***

To complement formal databases, the following approaches will also be employed:

- Backward citation tracking of included studies (reference list review)
- Forward citation tracking using Scopus and Web of Science to identify recent work citing included articles
- Manual hand-searching of key journals in global mental health and digital health
- Targeted Google Scholar searches for grey literature or preprints not indexed elsewhere

Direct contact with study authors may be made for clarification on intervention characteristics, population details, or outcome measures if essential data are missing or unclear during extraction. All searches will be documented in detail and managed using Rayyan for screening and Microsoft Excel for tracking study-level decisions.

### Search strategy

The search strategy for this systematic review will be developed using a structured combination of keywords and controlled vocabulary, such as MeSH terms, tailored to capture the core elements of the review question: population, intervention, context, and outcomes. The strategy will employ Boolean operators to link terms and will be customized for each database to reflect its unique indexing features and search architecture.

Searches will be conducted in five key sources of evidence: PubMed, Scopus, Web of Science, EBSCOhost, and PsyberGuide. PubMed will be used to identify biomedical and global health literature; Scopus and Web of Science will provide multidisciplinary coverage, including social and behavioral sciences; and EBSCOhost will allow access to psychology and allied health literature, including PsycINFO. PsyberGuide, a curated repository of digital mental health applications, will be included to capture real-world, commercially available interventions targeting mental health. The inclusion of PsyberGuide is justified on the basis that many digital mental health tools in use across Africa may not be indexed in traditional academic databases but may be accessible through public app registries or consumer health platforms. A structured search of PsyberGuide as of April 2025 will be conducted, and app names will be cross-referenced with empirical studies retrieved from bibliographic databases. Only apps that meet the inclusion criteria and are supported by peer-reviewed empirical evidence will be retained.

The search will include literature published between January 1, 2015, and April 3, 2025, to ensure the inclusion of both pre- and post-pandemic digital mental health interventions. All searches will be limited to studies published in English. In EBSCOhost, filters will be applied to restrict results to peer-reviewed journal articles and exclude non-empirical sources such as protocols, editorials, and opinion pieces. Additional database-specific filters, when available, will be applied to limit the results to human studies conducted with adolescent and young adult populations in African settings.

The search terms will cover a wide range of digital mental health modalities, including mHealth, eHealth, mobile applications, computerized cognitive behavioral therapy (CCBT), telepsychiatry, serious games, virtual and augmented reality, and artificial intelligence-based tools. These will be combined with terms related to mental health conditions and outcomes, such as depression, anxiety, psychological distress, stress, and psychosocial support. Population terms will focus on adolescents, young people, and individuals aged 10 to 24 years. Geographical terms will include the term “Africa” along with the names of all 54 African countries to ensure specificity. Terms related to feasibility, acceptability, implementation, effectiveness, engagement, and equity will be used to capture relevant implementation outcomes. Finally, study design filters will include terms such as randomized controlled trial, cohort study, quasi-experimental study, mixed-methods design, cross-sectional study, and other empirical formats. An example search strategy for PubMed in the data file ^39^.

## Study records

### Data management

All records retrieved through the search will be imported into Rayyan, a web-based application designed to facilitate collaborative screening of systematic reviews. Rayyan will be used to detect and remove duplicates, as well as to manage the title and abstract screening process. Following this, eligible full texts will be uploaded and tracked within the same platform. A Microsoft Excel spreadsheet will be used in parallel to organize data extraction, track inclusion decisions, document reasons for exclusion, and manage key variables across all included studies. This spreadsheet will serve as the master data extraction file and be backed up in secure cloud storage with controlled access by the review team.

### Selection process

Study selection will be conducted in three phases: title and abstract screening, full-text review, and final inclusion. Two reviewers (AGM and ND) will independently screen all titles and abstracts for relevance against the inclusion and exclusion criteria. Studies marked as potentially eligible by either reviewer will be retrieved for full-text review. The same reviewers will independently assess full-text articles, and any disagreements will be resolved through discussion or referral to a third reviewer (SN) for adjudication. Studies that meet eligibility criteria will proceed to data extraction and risk of bias assessment. A record will be kept for all excluded studies at the full-text stage, with reasons for exclusion documented.

### Data collection process

A standardized data extraction form will be developed and piloted on three studies to ensure clarity, consistency, and completeness. Once finalized, the form will be used by the two primary reviewers (ND and TS) to independently extract data from all included studies. The extracted data will be compared for discrepancies, which will be resolved through discussion or by consulting a third reviewer (SN) if needed. When necessary, attempts will be made to contact study authors to clarify missing or ambiguous information, particularly for population characteristics, intervention modalities, and mental health outcome measures. All extraction will be performed using Microsoft Excel with version control.

### Data items

The following data will be extracted from each study:

• **Study identification**: Author(s), year of publication, country or region, journal/source, and DOI or URL.

• **Study design and methods**: Study type (RCT, quasi-experimental, observational, etc.), sampling strategy, recruitment context, setting (community-based or remote), and duration of follow-up.

• **Population**: Age range, gender distribution, HIV status (if applicable), education level, socioeconomic characteristics, sample size, and inclusion/exclusion criteria.

• **Intervention characteristics**: Type of digital mental health intervention (e.g., mHealth, app-based, SMS, chatbot), delivery method, provider, and description of content or functionality.

• **Comparators**: Description of comparison group(s) if applicable, including active controls, passive controls, or no comparator.

• **Outcomes**: Mental health outcomes (including depression, anxiety, psychological well-being, and stress/emotional regulation), feasibility, engagement, equity, and other implementation metrics.

• **Equity dimensions**: Acceptability, gender inclusivity, financial and digital accessibility, language/cultural adaptation, and reach to marginalized populations.

• **Funding sources and ethical approvals**: Where reported, details of funders, ethical review boards, and trial registration numbers will be documented.

Where studies present data at multiple time points or across subgroups (e.g., by gender or HIV status), this information will be extracted separately.

### Outcomes and prioritization

The review will prioritize outcomes based on relevance to mental health and implementation science in digital contexts. Primary outcomes will include: reduction in depression and anxiety symptoms, improvement in psychological well-being, stress reduction, and emotional regulation. These outcomes were prioritized due to their frequency in adolescent mental health literature and relevance to the effectiveness of digital interventions.

Secondary outcomes will include feasibility (as defined by uptake, engagement, and usability), acceptability (user satisfaction, willingness to recommend), equity-related dimensions (gender inclusivity, financial accessibility, digital literacy), and retention or adherence to the intervention. Outcomes related to implementation such as cost, scalability, and provider burden will also be recorded when reported. The rationale for inclusion of secondary outcomes is to provide a comprehensive understanding of not only what works, but for whom and under what conditions, in line with the WHO evaluation framework for digital health interventions.

### Risk of bias in individual studies

Risk of bias will be assessed independently by two reviewers using the Cochrane Risk of Bias 2 (RoB 2) tool for randomized controlled trials and the ROBINS-I tool for non-randomized studies. Assessments will be made at both the study and outcome levels where applicable, particularly for mental health endpoints. Discrepancies in judgments will be resolved through discussion or referral to a third reviewer.

Key domains to be evaluated include selection bias, performance bias, detection bias, attrition bias, and reporting bias. For non-randomized studies, additional domains such as confounding and selection of participants will be considered. Where ten or more studies report on a common outcome, funnel plots will be used to assess the possibility of publication bias. Risk of bias judgments will inform the interpretation of findings and will be integrated into sensitivity analyses and assessments of evidence quality.

## Data

### Synthesis

Study data will be quantitatively synthesized if there is sufficient homogeneity in the characteristics of included studies, specifically in relation to participant age range (10–24 years), intervention type (e.g., SMS-based, app-based), outcome constructs (e.g., depression, anxiety), and measurement instruments. A meta-analysis will be conducted when three or more studies report on a comparable outcome using consistent or convertible metrics. Continuous outcomes such as symptom reduction scores will be summarized using either mean differences (MDs) when the same scales are used or standardized mean differences (SMDs) when different instruments are applied. Dichotomous outcomes such as adherence, satisfaction, or uptake will be synthesized using odds ratios (ORs) with 95% confidence intervals.

A random-effects model will be used for all meta-analyses to account for anticipated clinical and methodological heterogeneity across study settings and digital platforms. Heterogeneity will be assessed using the I ^2^ statistic, with values of 25%, 50%, and 75% indicating low, moderate, and high heterogeneity respectively. If sufficient data are available, Kendall’s tau will also be employed to explore correlations between study characteristics and effect sizes. Where appropriate, visualizations such as forest plots will be generated to support quantitative interpretation.

Additional analyses are planned to explore variations in outcome patterns. Subgroup analyses will assess whether studies conducted before and after 2020 differ in terms of outcomes, feasibility, and digital engagement, accounting for changes induced by the COVID-19 pandemic. Comparative analyses will also examine outcomes across different digital modalities—such as app-based, SMS-based, and gamified interventions—as well as across specific populations, including adolescents living with HIV, adolescent girls and young women, and urban refugee youth. Sensitivity analyses will be conducted by excluding studies with high risk of bias and those with small sample sizes (fewer than 30 participants), to test the robustness of the synthesis. If a sufficient number of studies are identified, meta-regression may be conducted to explore the relationship between implementation factors—such as duration, delivery format, and digital literacy assumptions—and reported mental health outcomes.

If the heterogeneity across studies is too high or if insufficient data are available to permit a reliable quantitative synthesis, a narrative synthesis will be undertaken. This synthesis will organize the evidence by type of digital mental health intervention, target population subgroup, delivery agent (e.g., peer, health professional), and implementation setting. Thematic matrices and visual aids such as heatmaps and bubble plots will be used to identify patterns, highlight gaps, and explore the extent to which interventions address health equity and accessibility in different contexts.

### Meta-bias(es)

To assess the presence of meta-biases, publication bias will be evaluated using funnel plots and Egger’s test where ten or more studies contribute data to the same outcome. The review will also assess selective outcome reporting by comparing prespecified outcomes in methods sections with those reported in study results. When protocols or trial registrations are available, these will be cross-checked against published articles to determine whether outcomes were selectively reported or omitted. Any discrepancies suggestive of reporting bias will be documented and used to inform the interpretation of findings and the overall GRADE assessments of evidence certainty.

### Confidence in cumulative evidence

The overall strength of the body of evidence for each prioritized outcome will be assessed using the GRADE (Grading of Recommendations, Assessment, Development and Evaluations) approach. This method offers a structured and transparent framework for rating the certainty of evidence across studies included in the review. GRADE assessments will be applied separately for each outcome domain, including mental health symptom reduction (such as depression, anxiety, and stress), psychological well-being, feasibility, acceptability, engagement, and equity-related considerations. Each outcome will be evaluated based on five core domains: risk of bias, inconsistency, indirectness, imprecision, and publication bias. Randomized controlled trials will initially be considered high-certainty evidence and may be downgraded depending on limitations in one or more of the domains. Non-randomized studies will begin at low-certainty and may be upgraded in specific circumstances, such as the presence of large effect sizes, dose-response gradients, or when all plausible confounders would reduce rather than explain the observed effect.

Risk of bias will be judged based on prior assessments using RoB 2 or ROBINS-I, depending on the study design. Inconsistency will be evaluated through the heterogeneity of results, quantified using I ^2^ statistics when meta-analysis is conducted. Indirectness will be considered in terms of the relevance of populations, interventions, comparators, and outcomes to the review objectives. Imprecision will be assessed through the width of confidence intervals and the overall sample size contributing to an effect estimate. Publication bias will be evaluated through visual inspection of funnel plots and comparison with expected outcomes where applicable.

Two reviewers (NW and TS) will independently conduct the GRADE assessments, and discrepancies will be resolved through discussion or consultation with a third reviewer (MN). A Summary of Findings table will be prepared for each main outcome, presenting the number of studies and participants, study design, direction and magnitude of effect, and the final certainty rating (high, moderate, low, or very low). The resulting GRADE assessments will guide the interpretation of the review findings and inform recommendations for future research and policy decisions regarding digital mental health interventions for adolescents and young people in African contexts.

## Data Availability

The completed PRISMA-P checklist supporting this protocol is openly available in the Africa Health Research Institute repository under the title “ *PRISMA-P Checklist for a Systematic Review Protocol on Digital Mental Health Interventions Among Adolescents and Young People (Aged 10–24 Years) in Africa"* ^39^. The dataset can be accessed at https://doi.org/10.23664/ahri.digital.mental.health.interventions. The dataset documents adherence to systematic review reporting standards, including administrative information, introduction, and methods sections. It is licensed under the Creative Commons Attribution 4.0 International (CC BY 4.0) license, permitting unrestricted use, distribution, and reproduction in any medium, provided the original work is properly cited.
