# Supplementary material for: Digital mental health interventions for adolescents and young people (10–24 years) in Africa: A protocol for a systematic review of mental health outcomes, engagement, and equity considerations
Source: Wellcome Open Res. 2025 Jul 4;10:331. [Version 1] doi: 10.12688/wellcomeopenres.24117.1 (PMC12405850; doi:10.12688/wellcomeopenres.24117.1)
Supplement: Peer review history

# Reviewer report: Reviewer response for version 1

**DOI:** 10.21956/wellcomeopenres.26606.r128047

**Published:** 2025-09-02

**Authors:** Shillah Mwaniga Mwavua

**Affiliations:** 1 Vrije University, Amsterdam, Netherlands Antilles

Introduction section 

 The introduction and objectives are generally strong, but a few improvements could enhance clarity and focus.

 1.Consider narrowing the discussion on digital health to focus specifically on mental health interventions, which will make the narrative more concise and directly relevant.

 2.The discussion of equity and the digital divide could be more organized by combining points on gender, socioeconomic status, digital literacy, and cultural attitudes, and grounding them in a clear framework such as PROGRESS-Plus.

 3.Consider, framing the objectives as specific research questions ,it will help clarify the review’s scope and make the aims more precise and actionable

 Methods section

1.  Group similar digital interventions and separate primary clinical outcomes from implementation outcomes for clarity.
2.  Specify how equity factors (e.g., gender, SES, digital access) and grey literature will be extracted and assessed.
3. Condense database and PsyberGuide details, clarify search terms/Boolean logic, and note handling of duplicates and language limits
4. Data section 1. Clarify roles of Rayyan and Excel, and specify how equity-related data will be extracted and linked to a framework like PROGRESS-Plus. 2. Separate clinical and implementation outcomes and explain how multiple time points or subgroups will be handled.3. Condense repetitive details on meta-analysis and heterogeneity, and clearly show how risk of bias feeds into GRADE assessments.

Is the study design appropriate for the research question?

Yes

Is the rationale for, and objectives of, the study clearly described?

Yes

Are sufficient details of the methods provided to allow replication by others?

Partly

Are the datasets clearly presented in a useable and accessible format?

Not applicable

Reviewer Expertise:

Mental health -Adolescents and postpartum women, low-cost interventions /digital interventions. Mental health Systems strengthening and capacity building.

I confirm that I have read this submission and believe that I have an appropriate level of expertise to confirm that it is of an acceptable scientific standard.

# Reviewer report: Reviewer response for version 1

**DOI:** 10.21956/wellcomeopenres.26606.r127324

**Published:** 2025-08-27

**Authors:** Isaiah Gitonga

**Affiliations:** 1 Maynooth University, Maynooth, County Kildare, Ireland

Overall, this is a clear and well-written protocol. Well done, this is much-needed research, particularly the focus on equity and implementation outcomes. There is a clear gap in that areas as it relates to DMHIs in this population.

 Some minor comments for consideration:

 Abstract

- In the *Methods* section, regarding study designs: rather than listing all study designs individually, would it be simpler to state that “ *all study designs will be included*,” unless you intend to exclude specific designs?
- In the *Methods*, the outcomes of interest include “ *engagement, feasibility, acceptability, and equity-related factors*.” Would these be considered *implementation outcomes*? If so, you might consider amending the title to include “implementation outcomes” in place of “engagement and equity considerations.” This is, of course, just a suggestion, particularly since I know you are keen to emphasise equity and outcomes.

Introduction: Well done. The research gap is clearly identified, and the rationale for this review is well articulated.

 Methods

- How will “equity” be defined and operationalised for the purposes of this review? For instance, in the context of DMHIs, the *digital divide* is likely to be a prominent factor. Is this being considered as part of the equity question/consideration? Considering it’s such a broad concept – I think that needs to be clear.
- Improvement in ‘psychological wellbeing (PWB)” is listed as one of the outcomes -You may want to operationalise this further, by stating clearly that you will include studies measuring PWB using established tools such as the *Ryff Scales of Psychological Well-Being*, and constructs related to PWB, such as anxiety, depression, or distress. I acknowledge  you have already stated that, but you could make it clearer,

All the best in the review.

Is the study design appropriate for the research question?

Yes

Is the rationale for, and objectives of, the study clearly described?

Yes

Are sufficient details of the methods provided to allow replication by others?

Yes

Are the datasets clearly presented in a useable and accessible format?

Not applicable

Reviewer Expertise:

Connected Health, Mental Health Research, Implementation science

I confirm that I have read this submission and believe that I have an appropriate level of expertise to confirm that it is of an acceptable scientific standard.
